# PINK1/PARKIN signalling in neurodegeneration and neuroinflammation

**DOI:** 10.1186/s40478-020-01062-w

**Published:** 2020-11-09

**Authors:** Peter M. J. Quinn, Paula I. Moreira, António Francisco Ambrósio, C. Henrique Alves

**Affiliations:** 1 Jonas Children’s Vision Care, and Bernard and Shirlee Brown Glaucoma Laboratory, Columbia Stem Cell Initiative, Departments of Ophthalmology, Pathology and Cell Biology, Institute of Human Nutrition, Vagelos College of Physicians and Surgeons, Columbia University, New York, NY USA; 2 Edward S. Harkness Eye Institute, New York-Presbyterian Hospital, New York, NY USA; 3 CNC - Center for Neuroscience and Cell Biology, University of Coimbra, Coimbra, Portugal; 4 Center for Innovative Biomedicine and Biotechnology (CIBB), University of Coimbra, Coimbra, Portugal; 5 Laboratory of Physiology, Faculty of Medicine, University of Coimbra, Coimbra, Portugal; 6 Coimbra Institute for Clinical and Biomedical Research (iCBR), Faculty of Medicine, University of Coimbra, Coimbra, Portugal; 7 Association for Innovation and Biomedical Research on Light and Image (AIBILI), Coimbra, Portugal; 8 Clinical Academic Center of Coimbra (CACC), Coimbra, Portugal

**Keywords:** PINK1, PARKIN, Mitophagy, Neurodegeneration, Alzheimer’s disease, Parkinson’s disease

## Abstract

Mutations in the PTEN-induced kinase 1 (PINK1) and Parkin RBR E3 ubiquitin-protein ligase (PARKIN) genes are associated with familial forms of Parkinson’s disease (PD). PINK1, a protein kinase, and PARKIN, an E3 ubiquitin ligase, control the specific elimination of dysfunctional or superfluous mitochondria, thus fine-tuning mitochondrial network and preserving energy metabolism. PINK1 regulates PARKIN translocation in impaired mitochondria and drives their removal via selective autophagy, a process known as mitophagy. As knowledge obtained using different PINK1 and PARKIN transgenic animal models is being gathered, growing evidence supports the contribution of mitophagy impairment to several human pathologies, including PD and Alzheimer’s diseases (AD). Therefore, therapeutic interventions aiming to modulate PINK1/PARKIN signalling might have the potential to treat these diseases. In this review, we will start by discussing how the interplay of PINK1 and PARKIN signalling helps mediate mitochondrial physiology. We will continue by debating the role of mitochondrial dysfunction in disorders such as amyotrophic lateral sclerosis, Alzheimer’s, Huntington’s and Parkinson’s diseases, as well as eye diseases such as age-related macular degeneration and glaucoma, and the causative factors leading to PINK1/PARKIN-mediated neurodegeneration and neuroinflammation. Finally, we will discuss *PINK1/PARKIN* gene augmentation possibilities with a particular focus on AD, PD and glaucoma.

## Background

Mitochondria, first discovered in the late 19^th^ century, are considered key for cellular bioenergetics [1, 2]. They consist of a double membrane with an intermembrane space. The inner membrane forms folds called cristae which provide an increased surface area for chemical and redox reactions to take place [3–5]. Mitochondria produce the majority of cellular adenosine triphosphate (ATP) through oxidative phosphorylation (OXPHOS). The protein complexes (cI-IV) of the respiratory chain transfer electrons from NADH and FADH_2_ (provided by the Krebs cycle) to molecular O_2_, a process also known as the electron transport chain (ETC). The ETC creates a membrane potential (ΔΨm) across the mitochondrial inner membrane by pumping protons from the mitochondrial matrix to the intermembrane space, thus creating a high concentration of protons in the intermembrane space and a low concentration in the mitochondrial matrix. Subsequently, along this chemiosmotic gradient, the protons move back into the mitochondrial matrix, via ATP synthase (cV). ATP synthase uses this process to create ATP from adenosine diphosphate (ADP) and inorganic phosphate (P_i_) [6–9].

Previously thought to be only the “powerhouse” of the cell it is now clear that mitochondria are multifaceted. In addition to their role in cellular bioenergetics, mitochondria control reactive oxygen species (ROS) levels and calcium homeostasis, and biosynthesize macromolecules including lipids, amino acids and nucleotides [10]. Furthermore, mitochondria are involved in many cellular physiological processes, including cell fate, differentiation, proliferation and apoptosis [11, 12]. Alongside its more established roles, mitochondria are key regulators of the innate and adaptive immune system. Immune cells undergo significant cell-type specific metabolic changes during an immune response, moving from a quiescent to an active state that requires significant metabolites from mitochondria [13, 14]. Mitochondria can regulate immunity via metabolic pathways, inducing transcriptional changes, activating inflammation, mitochondrial dynamics (fission and fusion) and endoplasmic reticulum signalling [14, 15].

Mitochondrial stress, either driven by the environment, pathogenesis or ageing, leads to a myriad of dysregulation that can cause both neurodegeneration and neuroinflammation. Mitochondria are vital in regulating cellular adaption to stressors, including impaired biogenesis, mitochondrial DNA (mtDNA) damage, ageing, nutrient restriction and aberrant imbalances between fission and fusion events. If left unchecked, these processes can cause damage to nucleic acids, lipids and proteins through ROS, resulting in sustained oxidative stress [16, 17]. Oxidative stress modulates mitochondrial dynamics through posttranscriptional modifications, including ubiquitination [18]. This, in turn, leads to a build-up of damaged mitochondria and ultimately causes cell death and broader tissue dysfunction. In particular, tissues with high energy demands such as the heart, muscles, brain and retina are susceptible to mitochondrial dysfunction [19]. To mitigate the effects of stressors, several control mechanisms can be activated contributing to mitochondrial homeostasis [17].

Mitochondria first-line defence mechanisms including enzymatic (such as superoxide dismutase, the peroxiredoxin/thioredoxin system and the glutathione peroxidase/reductase system) and non-enzymatic (such as GSH, vitamins E, A and C) antioxidants contribute to the maintenance of redox homeostasis [20]. However, beyond the utilization of antioxidants there exists several mitochondrial quality control mechanisms. These include regulation of mitochondrial fission and fusion events, which facilitate segregation of damaged mitochondria and axonal transport of mitochondria (fission) and the exchange of materials needed for their repair, such as mtDNA (fusion) [16, 21]. The mitochondrial unfolded protein response system, a mitochondria-to-nucleus transduction pathway, which promotes mitochondrial and cellular function if mitochondrial damage is sensed [22]. The ubiquitin–proteasome system leads to degradation of damaged outer mitochondrial membrane (OMM) proteins, and proteases lead to the removal of inner mitochondrial membrane (IMM) and mitochondrial matrix proteins [23]. Lastly, the export of damaged proteins via mitochondrial-derived vesicles (MDVs) or selective removal of damaged mitochondria via mitophagy end with their degradation in lysosomes [24].

PTEN-induced kinase 1 (PINK1) and Parkin RBR E3 ubiquitin-protein ligase (PARKIN) signalling play a key role in mitophagy and mitochondrial motility and size. PINK1 accumulates at the OMM in response to a reduction in mitochondrial ΔΨm caused by damage/dysfunction. In turn, this recruits PARKIN from the cytosol to the OMM were its E3 activity promotes mitophagy, through ubiquitination of mitochondrial proteins, leading to mitochondrial degradation. Defective mitophagy and PINK1/PARKIN signalling are present in neurodegenerative diseases including Alzheimer’s disease (AD), Parkinson’s disease (PD) and glaucoma [25–30].

**Fig. 1:**
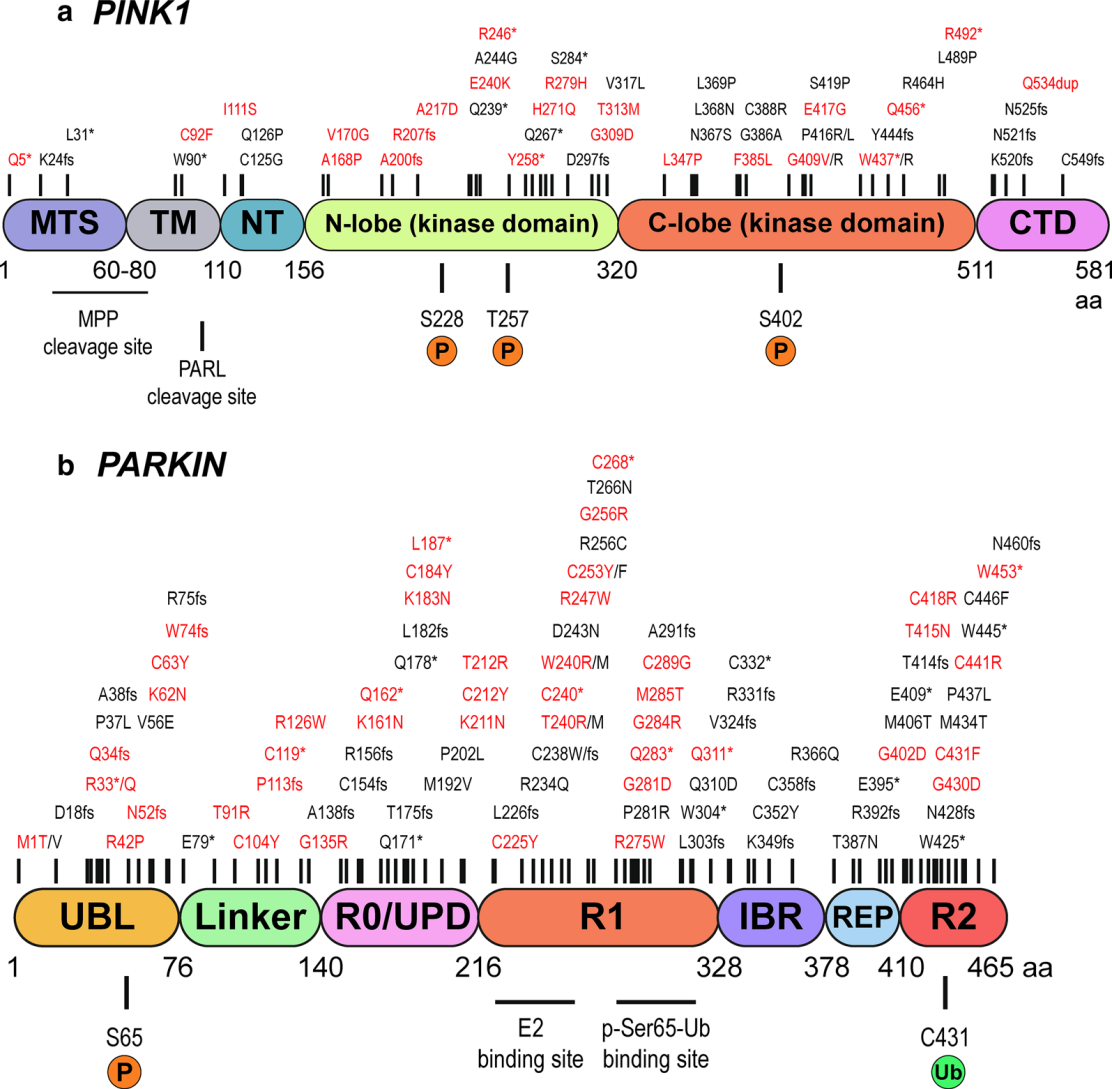
Schematic representations of PINK1 and PARKIN domains and disease-related mutations. **a** PINK1 is composed by 581 amino acids, encompassing the mitochondrial targeting sequence (MTS), transmembrane region (TM), N-terminal regulatory region (NT), N-lobe of the kinase domain, C-lobe of the kinase domain and the C-terminal domain (CTD). Mitochondrial processing peptidase (MPP) and presenilin-associated rhomboid-like (PARL) cleavage sites and PINK1 auto-phosphorylation sites are depicted in the figure (S228, T257, S402). **b** PARKIN is formed by 465 amino acids with a ubiquitin-like domain (UBL), linker, really-interesting-new-gene (RING)/unique Parkin domain (R0/UPD), RING1 (R1), in-between-RING (IBR), repressor element of Parkin (REP), and a RING2 (R2) domain. E2 co-enzyme and p-Ser65-Ub binding sites, as well as Ser65 phosphorylation and Cys431 catalytic sites, are displayed. Disease-associated mutations collected from the movement disorder society genetic mutation (www.mdsgene.org/) and ClinVAR (www.ncbi.nlm.nih.gov/clinvar/) databases are displayed on top of schematic representation. In red are depicted the mutations considered pathogenic

Mutations in the PINK1/PARKIN signalling pathway disrupts the sensitive homeostatic and quality control processes conducted by mitochondria. Mutations in PINK1 and PARKIN are localised throughout their genes affecting all their protein domains (Fig. 1). PINK1 and PARKIN mutations are responsible for more than 50% of the autosomal recessive juvenile parkinsonism (ARJP) cases [31]. However, there are several other causative genes for PD linked to mitochondrial dysregulation, including LRRK2, DJ1, ATP13A2 and SCNA, in addition to other PD risk genes [32–45]. Furthermore, dysregulation of PINK1/PARKIN signalling has been associated with amyotrophic lateral sclerosis (ALS) and Huntington’s disease (HD), as well as eye diseases, such as age-related macular degeneration (AMD), and is associated with retinal degeneration [46–51]. Efforts for the amelioration of mitochondrial dysfunction through lentiviral and adeno-associated viral (AAV) mediated PINK1 and PARKIN gene augmentation therapeutics show promise (Table 1).

**Table 1:** Summary of the *PARKIN* and *PINK1* gene augmentation viral vectors

Viruses	Capsid serotype	Promoter	Sequence	Injection place	Volume injected	Dose	Disease model	Animal	References
Lentivirus	HIV-1 based vector with VSVG envelops	PGK	Rat *Parkin*	S.N.	2.5 µl	3.6 × 10^8^ pg of p24 per ml	α-synuclein rat model for PD	Wistar rats	[52]
Lentivirus	HIV-1 based vector with VSVG envelops	CMV	Human *PARKIN*	S.N.	2 µl	10^8^ pg of p24 per ml	6-Hydroxydopamine rat model for PD	Rats	[53]
AAV	2/2	CBA	HA-tagged-*PARKIN*	S.N.	2 µl	3.6 × 10^12^ vg/ml	MPTP-treated mice, a model for sporadic PD	C57BL/6 mice	[54]
AAV	2/2 and 2/5	CMV/CBA	Human *PARKIN*	S.N.	2 × 2 µl	2.6 × 10^12^ vg/ml	6-Hydroxydopamine rat model for PD	Rats	[55]
AAV	2/2	CMV/CBA	Human *PARKIN*	S.N.	4 µl	5 × 10^12^ vg/ml	Tau-induced dopaminergic degeneration rat model for PD	Sprague–Dawley rats	[56]
AAV	2/6	PGK	Rat *Parkin*	S.N.	2 µl	4.7 × 10^10^ TUs/ml	Methamphetamine induced neurotoxicity rat model for PD	Sprague–Dawley rats	[57]
AAV	2/8	CMV	Human *PARKIN*	S.N.	2 µl in mice 3 µl in rats	2.0 × 10^11^ vg/ml	T240R-*PARKIN* induced dopaminergic degeneration model for PD	C57BL/6 J mice Wistar rats	[58]
AAV	2/2	CMV	Rat *Parkin*	Vitreous	5 µl	1.0 × 10^13^ vg/ml	Chronic hypertensive glaucoma model	Sprague–Dawley rats	[30]
AAV	2/1	CMV	Human *PARKIN*	Striatum	3 µl in rats 5 × 10 µl in monkeys	7.0 × 10^12^ vg/ml	α-synuclein rat model for PD	Sprague–Dawley rats Macaque monkeys	[59]
AAV	2/2	CMV	Human *PINK1*	Hippocampus	2 µl	5.0 × 10^12^ vg/ml	mAPP mouse model for AD	mice	[60]

*AAV* Adeno-associated virus, *CBA* hybrid cytomegalovirus immediate/early enhancer-chicken β-actin, *CMV* cytomegalovirus, *PGK* phosphoglycerate kinase, *S.N.* substantia nigra, *TU* transducing units, *Vg* viral genomes, *VSVG* vesicular stomatitis virus

## Main text

### PINK1/PARKIN signalling

**Fig. 2:**
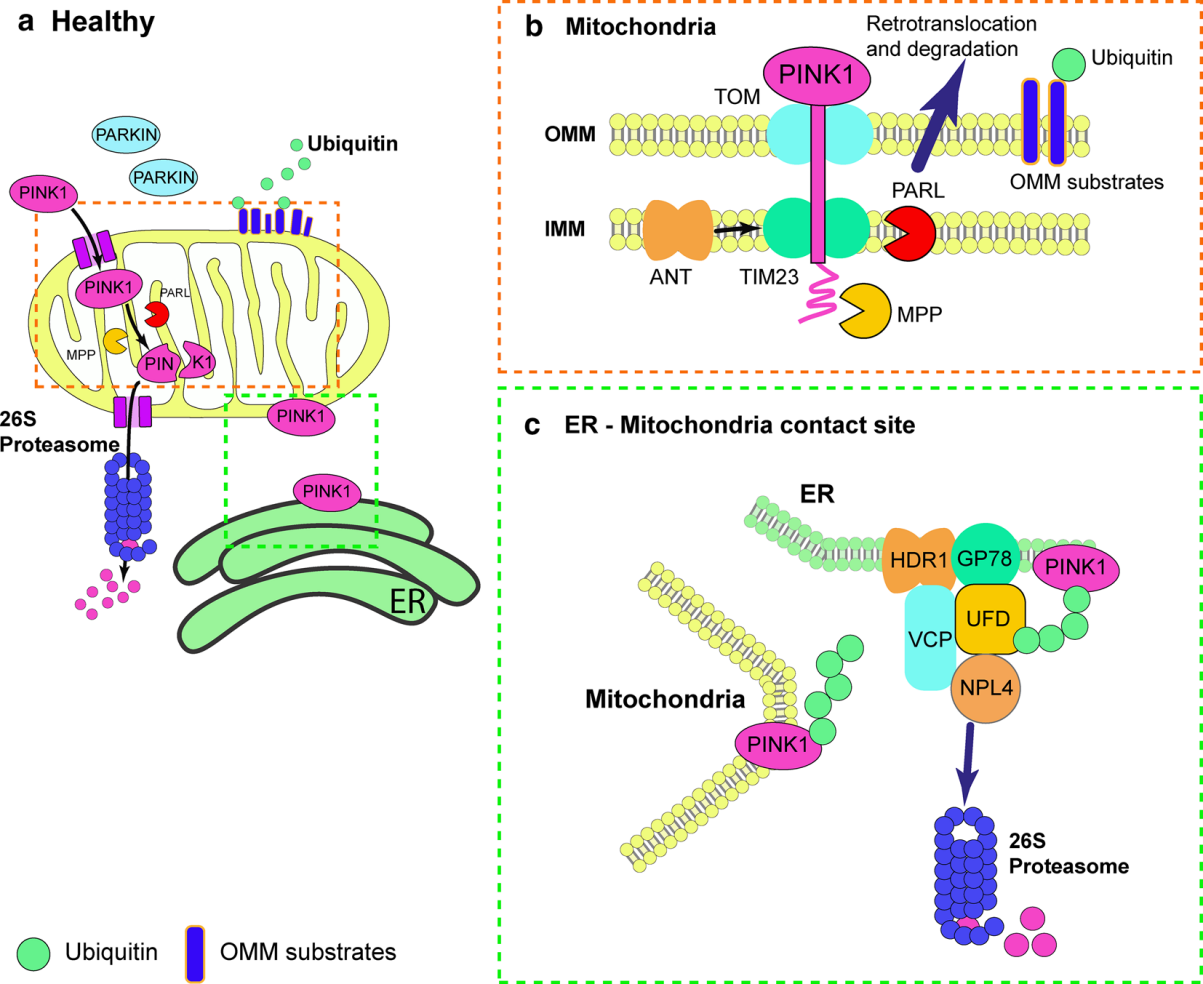
The canonical PINK1/PARKIN pathway. **a** and **b** In healthy mitochondria, PINK1 is constitutively imported via translocase of the outer membrane (TOM)/translocase of the inner membrane (TIM)23 complexes to the inner mitochondrial membrane (IMM), cleaved by two proteases (mitochondrial processing peptidase (MPP) and presenilin-associated rhomboid-like (PARL)) and retro-translocated to the cytosol. Cleaved PINK1 is then degraded by the ubiquitin/proteasome system. While Parkin remains inactive in the cytosol. (a and c) PINK1 is also present at the mitochondria-endoplasmic reticulum (ER) interface, where it interacts with the endoplasmic-reticulum-associated protein degradation (ERAD) machinery. At the ER, PINK1 degradation by the proteasome is controlled by the ERAD E3 ubiquitin ligases HRD1 and gp78 and by the ERAD-associated proteins VCP, UFD1, andUFD2A

**Fig. 3:**
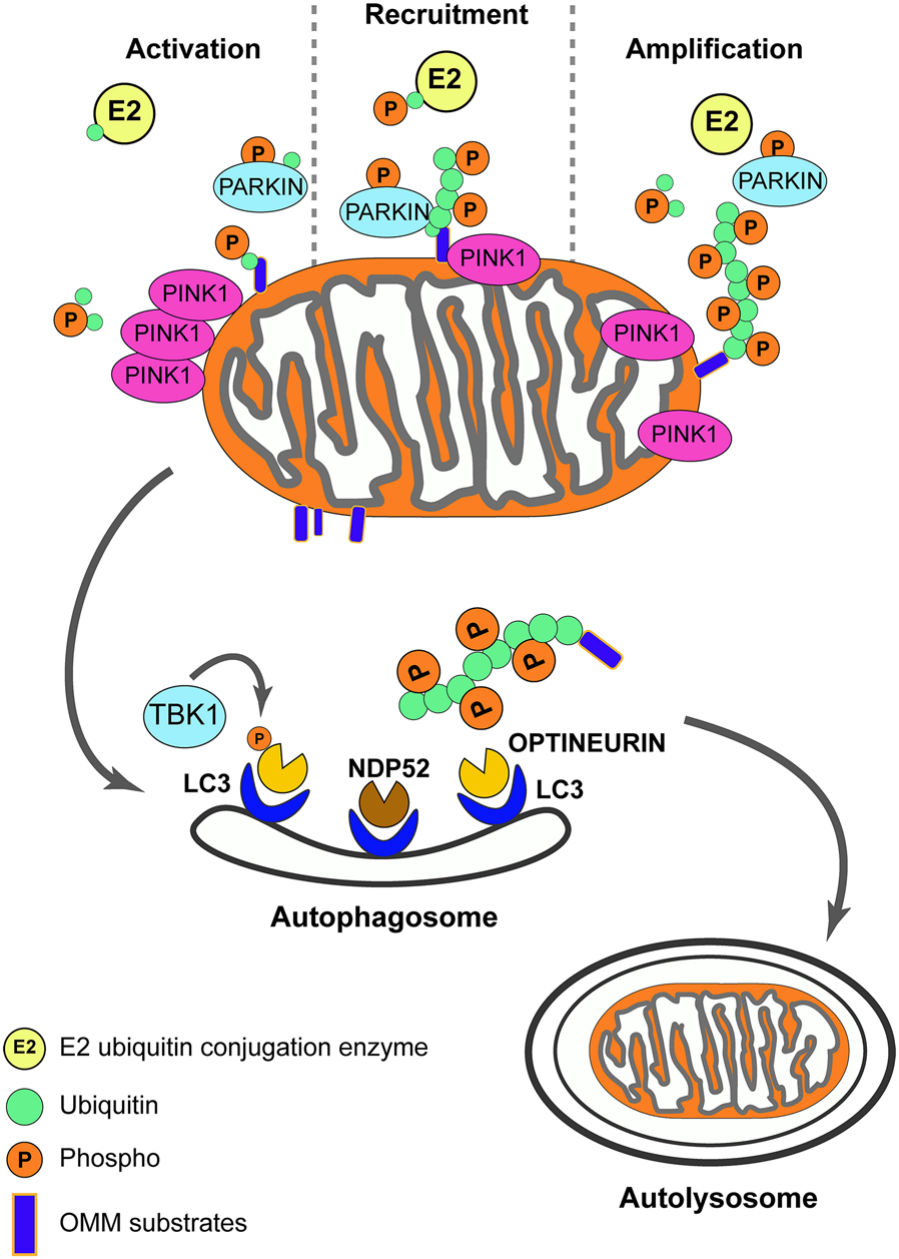
PINK1/PARKIN-directed quality control in damaged mitochondria. After damage, PINK1 is no longer imported into the inner mitochondrial membrane (IMM) and accumulates on the outer mitochondrial membrane (OMM). Here, a supercomplex composed by TOM complex subunits and PINK1 homodimers is formed, facilitating PINK1 autophosphorylation and activation. Once activated, PINK1 phosphorylates ubiquitinated substrates on the OMM and PARKIN enable its E3 ubiquitin ligase functions in concert with E2 ubiquitin-conjugating enzymes. PINK1-mediated phosphorylation of ubiquitin phospho-Ser65- ubiquitin on OMM substrates acts as the PARKIN receptor for its recruitment from the cytosol. PINK1 and PARKIN initiate a positive feedback loop, resulting in the coating of damaged mitochondria with phospho-ubiquitin chains. Individual OMM proteins decorated with poly-ubiquitin can be extracted from the membrane and degraded by the 26 S proteasome. Phospho-ubiquitin chains are bound by two mitophagy adaptors, nuclear domain 10 protein 52 (NDP52) and optineurin. Phosphorylation of optineurin by TANK Binding Kinase 1 (TBK1) enhances its binding to ubiquitin chains and promotes selective autophagy of damaged mitochondria. The two adaptors recruit autophagosomes via microtubule-associated protein 1A/1B-light chain 3 (LC3) binding, allowing the engulfment of dysfunctional mitochondria resulting in their direct degradation in lysosome

The mitochondrial serine/threonine-protein kinase PINK1, also known as BRPK and PARK6, protects cells from mitochondrial stress-induced dysfunction. Localized to chromosome 1 in position 1p36.12, the *PINK1* gene has 8 exons encoding a 581 amino acid protein. It contains an N-terminal mitochondrial targeting sequence (MTS), a transmembrane domain (TM), a N-terminal regulatory domain (NT), a conserved protein kinase domain comprising of a N-lobe and C-lobe, and lastly a C-terminal domain (CTD) (Fig. 1a). PARKIN, also known as PDJ, AR-JP, LPRS2 and PARK2 is localized to chromosome 6 in position 6q26 [61, 62]. *PARKIN* gene has 14 exons encoding a 465 amino acid protein which is comprised of an N-terminal ubiquitin-like (Ubl) domain and a C-terminal RING1-IBR-RING2 (RBR) domain. A RING0 domain sits N-terminally adjacent to RING1 and residing between the in-between-RING (IBR), and RING2 domains is a Repressor Element of Parkin (REP) motif (Fig. 1b) [63–66]. Under healthy conditions, mitochondria have an optimal, relatively high ΔΨm and will import, process and lead to the degradation of PINK1 (Fig. 2). However, under unhealthy conditions like oxidative stress, low ΔΨm causes PINK1 mitochondrial accumulation leading to PARKIN recruitment from the cytoplasm and initiation of autophagic degradation of the damaged mitochondria, the mitophagy pathway (Fig. 3) [67–70]. This pathway is governed by phosphorylation and ubiquitination, posttranscriptional modifications mediated by PINK1 and PARKIN, respectively.

The tightly regulated import to and subsequent proteolysis of PINK1 in the mitochondria leads to its processing from the full length 63 kDa protein precursor to the mitochondrial processing peptidase (MPP)-processed 60 kDa intermediated, to its final presenilins-associated rhomboid-like protein (PARL)-processed 52 kDa “mature” form (Fig. 2a, b) [71–73]. The translocase of the outer membrane (TOM) and of the inner membrane (TIM)23 complexes facilitate the importation of the PINK1 precursor via interaction with its MTS to the IMM. At the IMM, PINK1 undergoes cleavage at Ala103 by the protease PARL in a ΔΨm dependent manner. Under normal, healthy conditions PINK1 is imported and processed for degradation. In contrast, if ΔΨm dissipates, PINK1 remains localized to the OMM and is unable to be processed by PARL [72, 74]. Upon cleavage, PINK1 returns to the cytosol to be degraded by the ubiquitin–proteasome system by UBR1, UBR2 and UBR4 through the N-end rule pathway, leading to low levels of PINK1 (Fig. 2a, b) [75]. Recently Sekine et al. [76] found some PD related *PINK1* mutations, I111S, C125G and Q126P, affecting an evolutionary conserved negatively charged amino acid cluster motif that constitutes the C-terminal of the PINK1 TM, can still be imported even if ΔΨm dissipates. These mutants were found not to be cleaved by PARL but by the protease OMA1 at the IMM, suggesting that PINK1 did not accumulate on the damaged mitochondria’s OMM for initiation of mitophagy. However, these PINK1 mutants could lead to PARKIN recruitment under OMA1 suppression.

In contradiction to the degradation of the 52 kDa PINK1 by the N-end rule pathway is the finding by the Przedborski Lab that ubiquitinated PINK1 is mostly anchored to the OMM and not in the cytosol. Importantly, they identified that the N-terminal phenylalanine forming a proposed N-degron motif of PINK1 was not facing the cytosol but rather located inside the OMM, suggesting PINK1’s low mitochondrial levels are due to continuous ubiquitination and proteasomal degradation under healthy conditions [73]. Recently, the same team identified the mechanism by which PINK1 content is kept at low levels. They found that upon PARL-processing the 52 kDa PINK1 localizes at the mitochondrial-endoplasmic reticulum interface and can interact with ER-associated degradation pathway E3 ligases Gp78 and HRD1 (Fig. 2c). These facilitate PINK1’s ubiquitination allowing valosin containing proteins, UFD1 and UFD2A, to target PINK1 for proteasomal degradation [77]. Other proteases such as matrix-AAA and caseinolytic mitochondrial matrix peptidase (ClpXP) can cleave PINK1. These may coordinate with PARL to govern the stability and localization of PINK1 [71, 75]. In damaged mitochondria, TOM does not import PINK1, and it remains uncleaved at the OMM, where it undergoes dimerization and autophosphorylation (Fig. 3) [78, 79]. Interestingly, Sekine et al. [76] found that without the TOM complex accessory member Tom7 PINK1 was imported to depolarised mitochondria. Tom7 appears crucial in PINK1 OMM accumulation and also plays a role in PINK1 kinase activation for PARKIN recruitment. Phosphoglycerate mutase family member 5 (PGAM5) also binds PINK1 and is required for mitochondrial stabilisation of full-length PINK1 on the OMM upon mitochondrial depolarisation, preventing its cleavage by PARL at the IMM [80].

Under basal conditions, three factors have been identified which show that PARKIN’s protein-folding maintains PARKIN in an autoinhibited state: (1) inaccessibility of the E2-binding site on RING1 due to its occlusion by the REP domain [65]; (2) a conserved cysteine residue on RING2 (Cys431) is made inaccessible by RING0 [64, 65, 81]; (3) the Ubl domain inhibits parkin activity through the interface with RING1 and IBR domains [82–85]. The protein folding of PARKIN thus prevents the binding of Ub containing E2s to RING1 and the subsequent thiol-based transfer of Ub to the RING2 cysteine residue. The Cys431 residue is catalytic, being required for the ligase activity of PARKIN. The catalytic residue allows for the formation of an isopeptide bond between Ub and the lysine residue of the protein [64, 65, 86].

PINK1 is upstream of PARKIN and through the Ub/Ubl switch leads to activation of PARKIN by their phosphorylation at residue Ser65 (Fig. 3) [83, 84, 87–93]. Phosphorylation of Ubl increases PARKIN*’*s affinity for pUb. The binding of pUb to PARKIN enhances the rate at which PARKIN itself is phosphorylated by PINK1 [94, 95]. Specific phosphorylation of either Ub or Ubl leads to PARKIN activation; concomitant phosphorylation, however, leads to enhanced PARKIN activation [91, 92, 94]. Binding of pUb to PARKIN*’*s Ubl domain is essential for remodelling of and exposure of RING1 to the binding of the Ub containing E2s and is in line with previous computational analysis [83, 96, 97]. Ubl phosphorylation or binding of pUb to Ubl has also been shown to lead to local rearrangement of the IBR and its decreased affinity for the Ubl domain, revealing cryptic binding sites in a region called the Ubiquitin Binding Region (UBR) [85]. Three surface areas, UBR1, 2 and 3, that could interact with Ub were explored. Both UBR2 and UBR3 were needed for PARKIN activity. The IBR rearrangement in active PARKIN allows binding of Ub containing E2s to the binding site on RING1 while its Ub creates a bridge to the IBR of a neighbouring PARKIN molecule [85]. This association allows for the utilization of the RING2 catalytic domain of neighbouring PARKIN molecules [85]. In summary, pUb is important for dissociation and phosphorylation of PARKIN*’*s Ubl domain allowing its recruitment to the mitochondria. Subsequent PINK1 activation of PARKIN through Ser65 phosphorylation in the Ubl facilitates binding of E2 enzymes leading to PARKIN*’*s ligase activity. Several mutations exist throughout PARKIN, affecting its activity and stability (Fig. 1b) [98–100].

If there is severe mitochondrial dysfunction the amplified phospho-ubiquitin chains on the OMM signal the recruitment of autophagy adaptors such as nuclear dot protein 52 (NDP52) and Optineurin (OPTN). In turn, NDP52 and OPTN lead to the recruitment and activation of tank binding kinase 1 (TBK1), activated TBK1 phosphorylates OPTN stabilizing its binding at the phospho-ubiquitin chains [101]. Interestingly, PINK1/PARKIN-dependent mitophagy-induced sequestration of TBK1 leads to its removal from its physiological role at the centrosome causing G2/M cell cycle arrest. This highlights a possible role of PINK1/PARKINs in mitochondrial quality control before cell division takes place, preventing “unfit” mitochondria being passed on to daughter cells [102]. OPTN and NDP52 along with other autophagy adaptors lead to the recruitment of microtubule-associated proteins 1A/1B light chain 3 (LC3), which engage with the autophagosome. Migration and subsequent fusion of the autophagosome with the lysosome, which is modulated by the RAS-related GTP-binding (Rab) proteins, creates the autolysosome where the mitochondrial proteins are degraded and processed for recycling (Fig. 3). Initiation of autophagy has been found in the absence of LC3 via the Unc-51 like kinase 1 (ULK1) complex, which is comprised of ULK1, FAK family kinase-interacting protein of 200 kDa (FIP200), autophagy related gene (ATG)12 and ATG101 [103]. The ULK1 complex, which mediates autophagy in a nutrient-dependent manner, is recruited to ubiquinated cargo independently of AMPK by the cooperation of NDP52, and TBK1 [103]. Recently, Nozawa et al. [104] found that TBC1 domain family member 9 (TBC1D9), which is recruited to mitochondria via Ca2^+^-dependent Ub-binding, is essential for the activation and recruitment of TBK1 and therefore the subsequent recruitment of NDP52 and the ULK1 complex to damaged mitochondria.

## PINK1/PARKIN in neurodegeneration

Neurodegeneration corresponds to any pathological conditions, primarily affecting neurons [105]. Typically, neurodegenerative diseases are progressive disorders that lead to neuronal degeneration and cell death. The umbrella term “neurodegenerative diseases” includes conditions such as AD, PD, amyotrophic lateral sclerosis (ALS), Huntington’s disease (HD) and also eye diseases, such as age-related macular degeneration (AMD), glaucoma and a subset of inherited retinal dystrophies. Ageing is considered a primary risk factor in most neurodegenerative diseases [106]. Mitophagy increases in muscles and neurons during ageing but disruption of PINK1/PARKIN signalling abolishes this increase, hindering this crucial quality control mechanism and thus allowing the accumulation of harmful mitochondria [107–111]. Imbalances in mitochondrial fission and fusion are important for neuronal dynamics and are affected in neurodegeneration being linked to programmed cell death pathways [112]. PINK1 and PARKIN are essential in these processes interacting with fission/fusion machinery molecules such as fission protein Drp1 (dynamin-related protein 1) and fusion protein OPA1 (optical atrophy 1). Overexpression of *Pink1* or *Parkin* in rat hippocampal neurons leads to increased fission and can suppress a mitochondrial elongation phenotype caused by *Drp1* knockdown. A similar phenotype is caused by PINK1 inactivation, leading to increased fusion. Yu et al. [113] found that in dopaminergic neurons, similarly to hippocampal neurons, PINK1/PARKIN had a comparable influence on mitochondrial dynamics with tipping the fission/fusion balance towards more fission.

Alzheimer’s disease, the most common cause of dementia in the elderly, is a progressive neurodegenerative disease leading to memory deficits and cognitive decline, which in turn lead to behavioural and speech impairments. Ageing is the predominant risk factor with a prevalence of 10% for individuals over the age of 65 [114]. Pathologically, AD is hallmarked by the presence of amyloid plaques, mainly consisting of agglomerated amyloid-β (Aβ) peptides, and neurofibrillary tangles, mostly consisting of hyperphosphorylated tau, which are associated to cellular degeneration [115]. Another prominent hallmark of AD is the accumulation of dysfunctional mitochondria [116]. Robust induction of PARKIN-mediated mitophagy is found in human patients’ brains and in a human amyloid precursor protein (hAPP) transgenic mouse model of AD [28]. During disease progression, cytosolic PARKIN levels are reduced, leading to increased mitochondrial dysfunction [28]. Mitochondria from AD patients skin fibroblasts exhibited slower recovery of ΔΨm after insult [27]. Dysregulated protein levels of PARKIN and PINK1 were found in AD fibroblasts and brain biopsies. In both AD fibroblasts and hippocampal brain biopsies from Braak II-III stage patients, full length and cleaved PINK1 were increased. However, while PARKIN was diminished in the AD fibroblasts, it was found upregulated in Braak VI stage hippocampal brain biopsies. In AD fibroblasts, PARKIN recruitment after mitochondria depolarisation was found to be reduced, indicating defective mitophagy due to insufficient tagging of damaged mitochondria. Overexpression of *PARKIN* could compensate for the defective mitophagy in the AD fibroblasts [27]. Familial cases of AD are linked to autosomal dominant mutations of presenilin 1 (PSEN1). Both PSEN1 and PSEN2 are involved in a molecular cascade that modulates mitophagy via their control of *PINK1* transcription and function. Goiran et al. found that PARKIN upregulates PSEN1 promoter activation. In turn, control of γ-secretase activity, by PSEN1, targets APP leading to its fragmentation, yielding Aβ and the APP intracellular domain (AICD). Interaction of forkhead box O3a (FOXO3a) with AICD initiates *Pink1* transcription and AICD-mediated control of autophagic processes, which were found to be PINK1 dependent. As PINK1 recruits PARKIN to damaged mitochondria this highlights a feedback loop between the two genes that may become disrupted in neurodegenerative conditions [117, 118].

Parkinson’s disease is a movement disorder attributed to the loss of dopaminergic neurons in the substantia nigra. Motor symptoms include resting tremor, rigidity and bradykinesias, while non-motor symptoms include autonomic dysfunction, anxiety and sleeping problems. PINK1 and PARKIN are mutated in some forms of familial PD [119, 120]. *Pink1* and *Parkin* null *Drosophila* have learning and memory abnormalities and weakened circadian rhythms, in addition to underlying electrophysiological irregularities in clock neurons [121]. Late-stage PD patients can develop dementia with an accumulation of α-synuclein in Lewy bodies [41, 59, 122, 123]. Nitrosative stress is a key pathological hallmark in PD and aging. Nitric oxide-induced S-nitrosylation of PARKIN and PINK1 leads to compromised mitophagy and thus accumulation of damaged mitochondria [124–126]. One of the major causes of early-onset PD is due to loss-of-function mutations in genes including glucocerebrosidase (*GBA*), *RAB39B*, *DJ*-*1*, *PINK1* and *PARKIN* [25, 26, 127–130]. *Pink1* and *Parkin* KO mice show minimal signs of neurodegeneration but still provide valuable insights into possible mechanisms of action [131–135]. *Parkin* KO mice have an increase in extracellular dopamine concentration in the striatum, there is reduction in synaptic excitability in spiny neurons and dysfunction of the nigrostriatal pathway [131]. Another mouse model, presenting inactivated PARKIN due to a exon 3 deletion causing a premature stop codon, showed cognitive and motor deficits with inhibition of both amphetamine-induced dopamine release and glutamate neurotransmission [133]. Additionally, some mouse and rat *Parkin* KO models exhibit no neurodegeneration or any detectable neurochemical or pathological changes compared to wild type counterparts [135, 136]. This may be due to developmental compensation for PARKIN in these models. Due to the lack of neurodegeneration found in mouse KO *Parkin* models, Stephenson et al. [137] tried a novel approach by creating a double KO of *Parkin* and Parkin co-regulated gene (PACRG). *Parkin* and *PACRG* share a bidirectional promoter, with the transcriptional start sites being approximately 200 bp apart. However, no abnormalities of the dopaminergic system in the substantia nigra and no loss of neurons were found.

Analysis of PARKIN and its substrates has yielded possible PD associated neurodegenerative mechanisms. PARKIN mediates the ubiquitination and proteasome-dependent degradation of synaptotagmin-11 (Syt11) under normal conditions [138]. *Syt11* is a novel risk gene involved in PD whose accumulation in dopaminergic neurons due to PARKIN dysfunction inhibits endocytosis and hence dopamine release leading to neurotoxicity [40, 138]. Interestingly, Wang et al. [138] found that knockdown of Syt11 in *Parkin* knockdown background lead to the recovery of the dopamine release in the substantia nigra. PARKIN also mediates the ubiquitination and proteasome-dependent degradation of Zinc finger protein 746 (ZNF746, also known as PARIS) under normal conditions [139]. Accumulation of ZNF76 occurs due to PARKIN inactivation and is present in PD human brain samples [139, 140]. ZNF746 is a transcriptional repressor of peroxisome proliferator-activated receptor-gamma (PPARγ) coactivator-1α (PGC-1α) expression and its target gene nuclear respiratory factor 1 (NRF-1). In *Parkin* KO animals, dopaminergic neurons loss was found to be in a ZNF746-dependent manner with its overexpression leading to dopaminergic neuronal loss in the substantia nigra [139]. Recently, Brahmachari et al. [140] found that ZNF746 is a pivotal mediator of α-synuclein induced neurodegeneration affecting both dopaminergic and non-dopaminergic neurons. In α-synuclein overexpression mouse models c-Abl kinase phosphorylation of PARKIN led to the impairment of its activity and subsequent accumulation of ZNF746. Importantly, they found that ablation of ZNF746 leads to the rescue of the neurodegenerative phenotype observed in α-synuclein models of familial and sporadic PD [140]. PARKIN inactivation also leads to the accumulation of another one of its substrates, aminoacyl-tRNA synthetase complex interacting multifunctional protein-2 (AIMP2), found to be increased in *Parkin* KO mouse models and PD brain samples [140–143]. AIMP2 overexpression causes a progressive and degenerative loss of dopaminergic neurons due to Poly(ADP-ribose) polymerase-1 (PARP1) over activation. PARP1 inhibition in the AIMP2 overexpressed mouse model was protective and prevented degeneration of dopaminergic neurons [141].

Outside its role as an E3 ubiquitin ligase involved in mitophagy, PARKIN also has a role in transcriptional regulation (reviewed by Costa et al. [144]). As an example, PARKIN has been found to undergo nuclear translocation upon DNA damage where it may play a role in the transcriptional control of DNA repair mechanisms such as base and nucleotide excision repair and double strand break repair [145]. The transcription factor role of PARKIN therefore may act as a cellular defense mechanism against genotoxicity and suggests that DNA damage plays a pathogenic role in neurodegenerative disease such as PD [144, 145]. Recently, Shires et al. [146] have identified a role for nuclear PARKIN during hypoxia in activation of estrogen-related receptor α (ERRα), which is a transcription factor associated with mitochondrial metabolism and biogenesis. Interestingly, they also found that PARKIN mutants, *ParkinR42P* and *ParkinG430D,* are excluded from the nucleus and therefore unable to induce the transcription factor role of PARKIN. Therefore, the transcriptional roles as well as the mitophagic roles of PARKIN should be considered in PD as well as other neurodegenerative conditions.A *Pink1* KO mouse model in which the pathogenic patient mutation G309D was inserted into exon 5 presented mitochondrial dysfunction leading to defects in ATP generation along with a reduction in dopamine in the nigrostriatal projection with a concurrent reduction in locomotor activity, but again without neurodegeneration [132]. Generation of a further *Pink1* KO mouse, where exons 4–7 were deleted and consequently the majority of the kinase domain was removed, creating a nonsense mutation, caused impairment of dopamine release with striatal plasticity reduction. These impairments were rescued either in the presence of dopamine receptor agonists or due to stimulation of dopamine release, again highlighting the relevance of the nigrostriatal circuit [134]. In the same *Pink1* KO mouse model, it was shown that relocation of PARKIN to mitochondria induced by a collapse of Δψm relies on PINK1 expression [147]. In another *Pink1* KO mouse model, where exon 2 to exon 5 were replaced with a LacZ/Neo cassette, impaired dopamine release was also found. As compared to wild-type, dopamine from striatal slices of *Pink1* KO mice decreased in an age-dependent manner. Additionally, it was found an age-dependent decrease in basal oxygen consumption rates and ATP levels in *Pink1* KO mice, which suggests that decreased ATP generation may be the cause of the decreased dopamine release [148]. Recently, silencing of *Pink1* in cultured mouse hippocampal neurons caused a decrease in postsynaptic density proteins PSD95 and Shank as well as glutamate receptor subunit NR2B and mGluR5. Interestingly, the authors found changes in actin regulatory proteins RhoGAP29 and ROCK2 which were concurrent with changes in spine morphology. The changes in dendritic spines, showing increased thin density spines and reduced head size of stubby spines, may be a sign of presymptomatic changes that lead to neurodegeneration in PD [149]. In comparison, a *Pink1* KO rat model showed nigral neurodegeneration with 50% dopaminergic cell loss, an increase in striatal dopamine and serotonin content and significant motor deficits [136].

The inability of rodent models to recapitulate the severe neurodegeneration seen in PD patients may be due to low levels of PINK1, as has been identified in mice [150]. These studies also suggest there may be PINK1 independent mitophagy pathways yet to be eluded too. Recently, CRISPR/Cas9-mediated *Pink1* deletion in rhesus macaques triggered severe neurodegeneration of the cortex, striatum and substantia nigra, with several new-borns dying shortly after birth [151, 152]. These data suggest that in humans full PINK1 loss may lead to lethality in early development. Interestingly, a KO mouse model of the PINK1 OMM stabilisation protein PGAM5 leads to a more severe PD-like animal model than in *Pink1* KO mouse models. The *Pgam5* KO mice show a significant degeneration in dopaminergic neurons in addition to a PD-like movement disorder characterised by gait changes and bradykinesia [80].

Lastly, in light of mitochondria’s role in the immune system, we should look to reassess the many disorders associated with defective mitochondrial genes in terms of potential autoimmunity. PD, as one example, has been recently hotly debated as also being an autoimmune disease [153–158]. PINK1 and PARKIN have been found to regulate adaptive immunity, being key for mitochondrial antigen presentation in a mitophagy independent process. This process instead relies on the generation of MDVs with a direct correlation between the extent of MDV formation and the amount of mitochondrial antigen presentation. PINK1 and PARKIN inhibit this process, the presence of PARKIN was found to be key in preventing Snx9 being recruited to mitochondria and initiating MDV formation [159]. Further supporting this notion, it was recently found that intestinal infection of *Pink1* KO mice with Gram-negative bacteria elicited mitochondrial antigen presentation and autoimmune mechanisms. These responses triggered mitochondrial-specific CD8^+^ T-cells that were found to induce dopaminergic neuron death. The infected *Pink1* KO mice presented acute motor symptoms [153]. Therapeutics that influence mitochondrial immune regulation will be an exciting area to be developed in treating these diseases.

Amyotrophic lateral sclerosis is a progressive and debilitating neuromuscular disease marked by degeneration of motor neurons in the brain and spinal cord, leading to muscle atrophy, paralysis and to death 3–5 years after disease onset. Mitochondrial dysfunction has been associated with ALS, with causative genes including autophagy adaptors OPTN and SQSTM1, and autophagy enhancer TBK1 [160–163]. Altered expression levels of mRNA and protein for PINK1 have been identified in human ALS patients muscle [164]. Mutations in superoxide dismutase 1 (SOD1) gene are associated with familial ALS [165]. A SOD1^G93A^ ALS mouse model exhibits dysregulated PINK1 and PARKIN and progressive defects in mitochondrial function and dynamics [47, 164]. In spinal cord motor neurons of the SOD1^G93A^ mouse model increased mitophagy, as marked by a mitochondrial accumulation of OPTN and SQSTM1, was found, while there was a depletion of PARKIN and mitochondrial dynamic and biogenesis proteins. Interestingly, *Parkin* overexpression in NSC34 motor neuron-like cells, in which human G93A mutant SOD1 was expressed, was found to exacerbate the effects of mitochondrial damage leading to increased cell toxicity. However, *Parkin* knockout (KO) in SOD1^G93A^ mice led to delayed disease progression with slower motor neuron loss and muscle denervation. Thus, chronic PARKIN expression in ALS may lead to sustained activation of mitochondrial quality control leading to a depletion of mitochondrial dynamic-related proteins and inhibition of mitochondrial biogenesis, and these alterations ultimately lead to progressive mitochondrial dysfunction [47].

A hallmark of ALS is the accumulation of transactive response DNA-binding protein 43 kDa (TDP-43) at ubiquitin-positive inclusions, and these TDP-43 protein inclusions have reduced PARKIN protein levels [166, 167]. PINK1 and PARKIN are differentially misregulated at the RNA and protein levels in animal models of TDP-43 proteinopathy. These models showed a decrease in *Parkin* mRNA and protein levels upon overexpression of TDP-43 but not PINK1. TDP-43 was found to govern *Parkin* mRNA levels in both an intron-mediated and intron-independent manner. While TDP-43 did not regulate *Pink1* at the RNA level, its overexpression led to the cytosolic accumulation of cleaved PINK1 due to the impairment of the ubiquitin–proteasome system [46]. In stress conditions, such as ageing, this accumulation of cleaved PINK1 leading to reduced mitochondrial activity may be a risk factor promoting neurodegeneration. Lastly, Sun et al. [46] found that by ameliorating the misregulation of PINK1 or PARKIN by their down or up-regulation, respectively, leads to suppression of the degenerative phenotypes observed in a TDP-43 proteinopathy fly model.

Huntington’s disease is a fatal autosomal dominant disorder caused by misfolding and aggregation of the huntingtin (HTT) protein due to expansion of a polyglutamine tract (CAG repeats) within its N-terminal domain. The disease leads to cognitive deficits, choreatic movements and psychiatric disturbances [168, 169]. The mutant HTT protein has been found to negatively affect the initiation of autophagy/mitophagy through interfering with the formation and stability of the ULK1 and PtdIns3K complexes, which are essential for autophagosome formation [170]. Mitochondrial fragmentation is a hallmark of HD patients with mutant HTT found to abnormally interact with fission protein Drp1 [171–174]. Additionally, swollen/degenerated mitochondria have been identified in a HD knock-in pig model which exhibited selective degeneration of striatal medium spiny neurons [175]. Furthermore, HD patients have impairment in the mitochondrial respiratory chain [176, 177]. In a *Drosophila* model of HD, mutant HTT led to mitochondrial fragmentation in photoreceptors, being abnormally ring-shaped. However, *PINK1* overexpression enhanced mitochondrial quality control in a PARKIN-dependent manner, alleviating the formation of the ring-shaped mitochondria. Additionally, they found that PINK1 neuroprotection in the *Drosophila* brain led to normalization of ATP levels, improved neuronal integrity and increased cell survival. Lastly, Khalil et al. [48] found that defective mitophagy found in striatal cells from a HD knock-in mouse could be partially restored upon *PINK1* overexpression.

Age-related macular degeneration is a complex retinal disorder and the leading cause of severe blindness in the elderly population, resulting from both environmental and genetic risk factors [178–180]. AMD affects central vision and its pathobiology includes activation of the innate immune response, neovascularisation, oxidative stress and a build-up of proteins and lipids [179, 181]. Accumulation of mtDNA damage is associated with AMD progression [182]. In the RPE of a Nuclear factor erythroid 2-related factor 2 (*NFE2L2/NRF2*) and peroxisome proliferator-activated receptor-gamma captivator 1-alpha (*PGC*-*1α*) double knockout (dKO) dry AMD-like mouse model, elevated levels of oxidative stress markers, damaged mitochondria, accumulated lysosomal lipofuscin and extracellular drusen-like deposits were found. Nrf2 is part of the Keap1-Nrf2 pathway which is important in oxidative stress regulation, and PGC-1α is involved in mitochondrial biogenesis and in the antioxidant defence system [183]. Recently, in the same *NRF2/PGC*-*1α* dKO mouse model at 1 year of age, dysregulation of mitophagy was evaluated. Compared to wild type RPE a significant increase in PINK1 and PARKIN levels on damaged mitochondria was found in the dKO, this additionally corresponded to an increase in the number of autophagosomes with mitochondrial cargo. However, despite elevated mitophagy initiation this model seemed to have uncompleted degradation of mitochondrial cargo via an unclarified dysfunction in the autolysosomes [49]. Mitophagy may be a novel therapeutic target for the amelioration of AMD. In a *Drosophila* model of calcium cytotoxicity in which active TRPP^365^ channels lead to retinal degeneration, abnormalities in mitochondrial morphology and function were found in photoreceptors. Interestingly, overexpression of both *PINK1* and *PARKIN* prevented the TRPP^365^-induced photoreceptor cell degeneration [51]. Moreover, in a PINK1/PARKIN-induced photoreceptor degeneration model, the induction of cell death by PINK1/PARKIN was found to be independent of mitophagy [50].

Glaucoma, caused by progressive degeneration of retinal ganglion cells, leads to severe and irreversible blindness, with 111.8 million people predicted to be affected by 2040 [184, 185]. Elevated intraocular pressure (IOP) is considered a major risk factor for glaucoma [186]. Therapies directed at lowering IOP have proved to be successful at preserving vision in some glaucoma patients, but this does not work for all patients [187]. Glutamate excitotoxicity, a pathophysiological mechanism in glaucomatous neurodegeneration, leads to changes in mitochondrial dynamics, causing their dysfunction and cell death [188]. Overexpression of *Parkin* protects retinal ganglion cells from glutamate excitotoxicity [189]. Furthermore, in a chronic hypertensive glaucoma rat model, overexpression of *Parkin* was protective, partially restoring mitophagy and improving mitochondrial health [30]. Recently, Chernyshova et al. [190] explored the role of glaucoma specific OPTN gene mutations and their effect on PARKIN-dependent mitophagy using mitophagy impaired HeLa cells. OPTN is a receptor for PARKIN-mediated mitophagy pathway, and mutations of *OPTN* cause primary open-angle glaucoma (POAG) [29, 191]. Interestingly, Chernyshova et al. [190] observed that while two ALS OPTN mutant proteins failed to rescue the impaired HeLa cells, seven glaucoma specific *OPTN* mutations did restore mitophagy and localized correctly to mitochondria. This work suggests that *OPTN* gene mutation in glaucoma may be mitophagy independent.

## PINK1 and PARKIN in neuroinflammation

Neurodegeneration and neuroinflammation are concurrent processes in many disorders. Neuroinflammation is a process that involves the synthesis and release of pro-inflammatory mediators, such as cytokines and chemokines, and infiltration of immune cells that if uncontrolled contribute to neurodegeneration exacerbation. Here, we summarize the supporting pieces of evidence for the involvement of PINK1 and PARKIN in neuroinflammation.

As discussed before, mutations in *PARKIN* and *PINK1* cause early-onset PD [25, 192]. Primary human blood-derived macrophages obtained from PD patients with *PARKIN* mutations display high levels of NLRP3 and IL-1β when stimulated with lipopolysaccharide (LPS)-nigericin or LPS-ATP [193]. *PINK1G309D*, the loss-of-function mutation associated with early-onset familial PD, promotes the expression of VCAM-1 and exacerbates the attachment of monocytes to brain endothelial cells [129]. Humans with monoallelic and biallelic *PARKIN* mutations display elevated serum levels of IL-6, IL-1β, CCL2 and CCL4, whereas the levels of these molecules in serum of *PINK1* heterozygotes were similar to those in control serum [194]. In contrast, mice lacking either *Pink1* or *Parkin* have no substantial PD-relevant phenotypes, and their levels of cytokines in the serum is unaltered [131, 134, 135, 194]. However, acutely prepared cortical slices from *Pink1* knockout mice, presented elevated levels of pro-inflammatory cytokines, such as TNF-α, IL-1β, and IL-6 [195]. In mature zebrafish systemic administration of LPS results in increased *Pink1* gene expression in the brain [196].

**Fig. 4:**
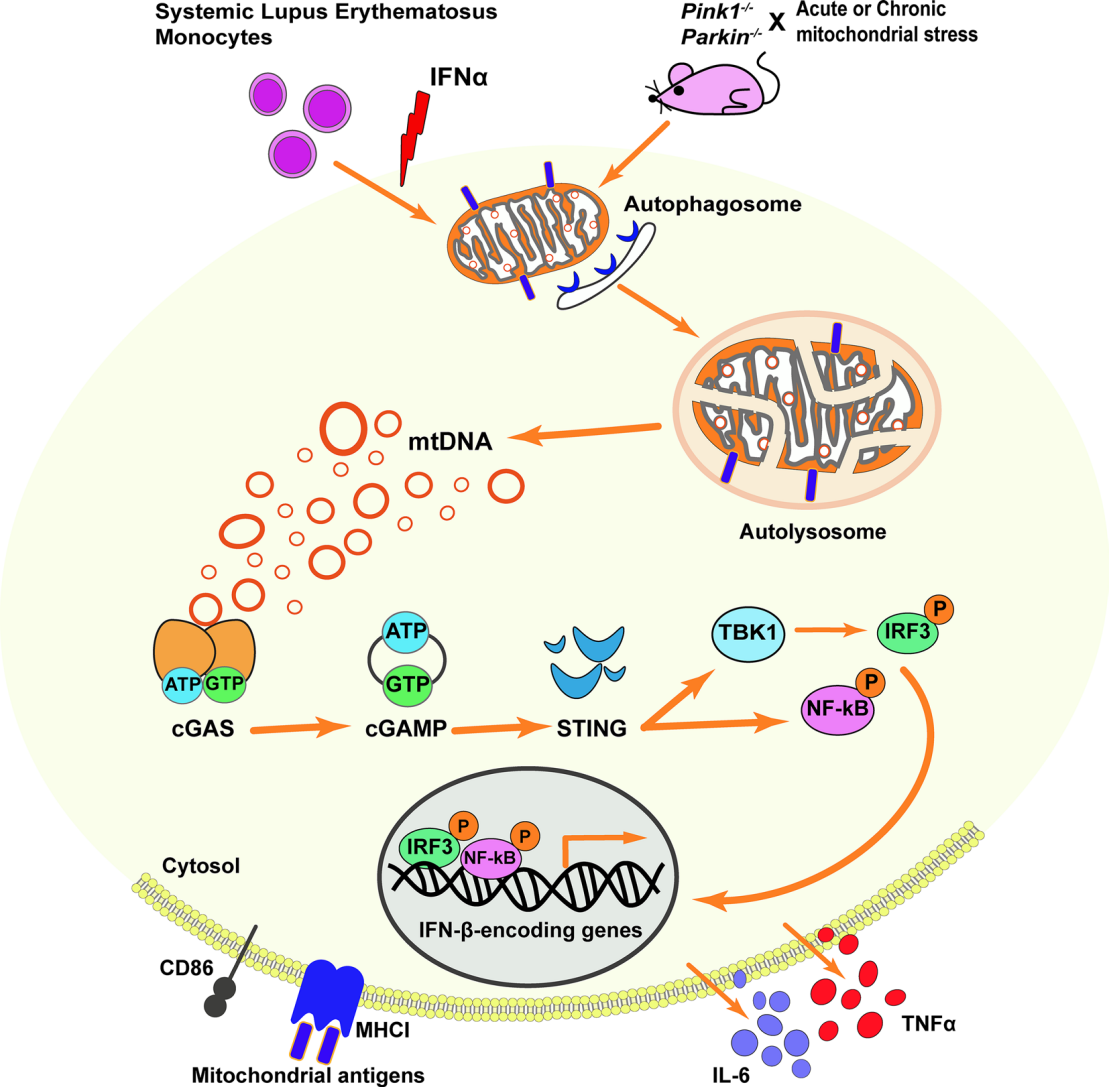
PINK1/PARKIN-signalling and inflammation. Mice lacking *Parkin* or *Pink1* upon acute (exhaustive exercise-induced) or chronic (mitochondrial DNA (mtDNA) mutation-induced) mitochondrial stress present inflammation due to the activation of the stimulator of interferon genes (STING) as result from the accumulation of mtDNA mutations and release of mtDNA into the cytosol. While, in systemic lupus erythematosus excessive IFNα damages mitochondrial respiration, leading to oxidative stress that impairs lysosomal degradation and obstructs autophagic clearance. Undegraded mtDNA from mitochondria, interact with the cytosolic DNA sensor cGAS in a sequence-independent way, promoting a conformational change of cGAS to catalyse the formation of 2,3-cyclic GMP-AMP (cGAMP). The cGAS activation, as well as cGAMP synthase, activate STING, recruiting binding kinase 1 (TBK1) as well as interferon regulatory factor 3 (IRF3). The IRF3 then displaces to the nucleus and induces immune-stimulated genes and type I IFN expression. The nuclear factor kappa-light-chain-enhancer of activated B cells (NF-κB) signalling can also be activated by STING. In the absence of PARKIN and PINK1, high levels of mitochondrial antigens are presented to major histocompatibility complex (MHC) class I molecules in macrophages and dendritic cells triggering an adaptive immune response

In mice lacking *Parkin* or *Pink1* upon both acute (exhaustive exercise-induced) or chronic (mtDNA mutation-induced) mitochondrial stress, a robust inflammatory phenotype is observed [194]. Following exhaustive exercise, *Pink1*^+*/*−^ mice show increased IL-6, IFNβ1, IL-12(p70), CXCL1 and CCL4, whereas *Parkin*^+*/*−^ mice display increased IL-6. Mice expressing a proofreading-defective mtDNA polymerase (mutator mice) accumulate mutations in mtDNA but do not exhibit neurodegeneration or elevated cytokines [194, 197]. However, PARKIN-deficient mutator mice presented elevated IL-6, IFNβ1, TNFα, IL-1β, CCL2, IL-12(p70), IL-13, IL-17, CXCL1 and CCL4 [194]. Inflammation derived from either exhaustive exercise or mtDNA mutation results from the activation of the stimulator of interferon genes (STING), a central regulator of the type I interferon response to cytosolic DNA, and not due to activation of NRLP3 [194] (Fig. 4). Interestingly, PARKIN-deficient mutator mice exhibit dopaminergic neuron loss and motor impairment that can be rescued by treatment with levodopa [109] and, as well, by loss of STING, by crossing PARKIN-deficient mutator mice with STING-null mice (goldenticket mice) [194]. STING is activated when double-stranded DNA binds cyclic guanosine monophosphate (GMP) - adenosine monophosphate (AMP) synthase (cGAS), which in turn generates cyclic GMP-AMP (cGAMP) [198]. PARKIN-deficient mice subjected to acute or chronic mitochondrial stress displayed both increased mtDNA copy number and ratio of mitochondrial to nuclear DNA in the serum; this increase is not rescued by loss of STING [194]. STING activation by binding of cGAS to cytosolic double-stranded DNA (dsDNA), including mtDNA, and STING-mediated inflammation resulting from an accumulation of mtDNA mutations in mutator mice, indicate that mtDNA is a crucial inflammatory signal in the absence of PARKIN [194]. Release of mtDNA into the cytosol, subsequent interaction of mtDNA with cGAS, and induction of IFNβ expression is also observed in mouse models of macular degeneration [199] and upon herpes virus infection [200]. Surprisingly, Whitworth and colleagues showed that knockdown of *Sting* or its downstream effector Relish using RNAi (in vivo), is insufficient to suppress the locomotor deficits or mitochondrial disruption in *Pink1* or *Parkin Drosophila* mutants [201]. Furthermore, *Sting* loss does not affect the behavioural phenotypes associated with a *Drosophila* mtDNA mutator model, nor the combined effect of mtDNA mutations in a *Parkin* background, concluding that phenotypes associated with loss of *Pink1/Parkin* are not universally due to aberrant activation of the Sting pathway [201]. Not only dysregulation of mitochondrial function promotes inflammation, but also inflammation itself leads to mitochondrial dysfunction suggesting the existent of a pro-inflammatory loop with mitochondria playing a central role. IFNα-mediated deregulation of mitochondrial metabolism, including mitochondria hyperpolarization and upregulation of PINK1, and impairment of autophagic degradation, results in cytosolic accumulation of mtDNA passible of being sensed via STING to promote further inflammation [202] (Fig. 4). *Parkin* knockout mice submitted to chronic LPS exposure develop fine-locomotor deficits and loss of nigral dopaminergic neurons. However, in these mice, neuroinflammatory responses in the midbrain are similar to the ones observed in wild-type mice [203].

In the absence of PARKIN and PINK1, high levels of mitochondrial antigens are presented by major histocompatibility complex (MHC) class I molecules in both macrophages and dendritic cells through mitochondrial-derived vesicles triggering adaptive immune response [159]. Therefore, PINK1 and PARKIN seem to repress mitochondrial antigen presentation providing a link between mitochondrial dynamics and the potential engagement of autoimmune mechanisms in the aetiology of PD [159].

The expression of PINK1 and PARKIN is increased in reactive astrocytes in the diseased human brain [204, 205], suggesting that these proteins affect or regulate glia-dependent immune responses. Lack of PINK1 increases glia-mediated primary neuron apoptosis and nitric oxide (NO)-dependent neuroblastoma cell death [206], suggesting that PINK1 in glial cells promotes a neuronal protective effect. Ablation of PINK1 differentially affects inflammation-induced gene expression and NO production in astrocytes, microglia and mixed astrocytes/microglia [206]. PINK1-deficient astrocytes show proliferation defects, increased p38MAPK activation [207], elevated NO production, impaired mitochondrial function and increased cytoplasmatic and mitochondrial ROS levels [206]. PINK1-deficient astrocytes exposure to LPS and IFNγ overexpress inducible nitric oxide synthase (iNOS), NO and TGFβ1. However, PINK1-deficient microglia only show decreased IL-10 secretion [206]. In vitro, LPS-activated murine microglia cell line (BV2) with reduced levels of PARKIN show increased levels of TNFα, IL-1β, IL-6 and iNOS mRNA via NF-κB and activating protein 1 (AP-1). Quite similar pro-inflammatory profile, with an increase of TNF-α, IL-1β, IL-6, IL- 18, monocyte chemoattractant protein-1 (MCP-1) and NRLP3 is also observed in *Parkin*-null primary microglia cells exposed to LPS [193]. Mouse microglia primary cultures, with reduced levels of PARKIN, present a similar increase in TNFα, IL-6 and iNOS and a decrease in IL-1β, after exposure to either IFNγ, TNFα or both [208]. These data suggest that PINK1 or PARKIN loss exacerbates inflammation and promotes survival of activated microglia, contributing to neuroinflammation. Furthermore, in macrophages, PARKIN suppresses LPS-induced expression of TNFα, IL-6 or MCP-1 production [209, 210].

### PARKIN and PINK1 gene augmentation therapy for neurodegenerative disorders

In the previous sections, we summarized the importance of PINK1 and PARKIN in controlling critical cellular mechanisms. The extensive published data pinpoint that disruption of PINK1/PARKIN signalling culminates in impaired mitochondrial function and ultimately contribute to neurodegenerative and neuroinflammatory processes. Thus, *PARKIN* and *PINK1* gene augmentation therapy seems, at least in theory, a promising strategy for brain and retinal degenerative disorders. Table 1 summarizes the viral vectors used in each study.

Pre-clinical studies show that *PARKIN* gene augmentation ameliorates disease features in several disease models [30, 52–59]. Amongst the different gene augmentation therapy vectors, lentiviral [52, 53] and adeno-associated viral (AAV) vectors have been described [30, 54–59]. Lentiviral-mediated gene therapy delivery of *Parkin* into substantia nigra significantly reduces α-synuclein-induced neuropathology, including preservation of tyrosine hydroxylase-positive cell bodies in the substantia nigra and sparing of tyrosine hydroxylase-positive nerve terminals in the striatum [52]. Moreover, overexpression of human *PARKIN* in rat’s substantia nigra prevented 6-hydroxydopamine-induced degeneration of dopaminergic terminals and cell bodies and ameliorated the motor behaviour [53]. In the recent years, AAV vectors have become popular gene delivery tools due to their safety profile, low immunogenicity, lack of toxicity and to the fact of the AAV genomes do not integrate into the host genome [211]. Moreover, the existence of several natural AAV serotypes and derivatives that differ in their tropism, makes AAV a powerful tool for gene delivery in the central nervous system. Several AAV serotypes including 2, 5, 6 and 8 have been used to transduce neurons and deliver *Parkin* under the control of the cytomegalovirus (CMV), CMV enhancer/chicken β-actin or phosphoglycerate kinase 1 (PGK) promoter [30, 54, 55, 57, 58]. As observed for lentiviral gene therapy vectors, AAV-mediated delivery of *Parkin* into the substantia nigra also demonstrated to improve disease features in different PD animal models. The therapeutic potential of AAV-gene transfer of *Parkin* on the dopaminergic system was assessed on 1-methyl-4- phenyl-1,2,3,6-tetrahydropyridine (MPTP)-treated mice, a model for PD [54]. AAV2/2-*Parkin* treatment resulted in a higher survival rate of dopamine neurons in the substantia nigra. Protection at the neuronal level was supported by increased amphetamine-induced contralateral turning behaviour, a test to evaluate presynaptic neurotransmission, once amphetamine inhibits the dopamine transporter and stimulates dopamine release from presynaptic axon terminals [54]. Another study tested the effects of AAV2/5-*Parkin* delivery before a 4-site striatal 6-hydroxydopamine lesion [55]. Parkin treated lesioned rats displayed 67% in amphetamine-induced rotational behaviour reduction and used their affected paw nearly twice as often as control rats in the cylinder test, demonstrating a clear motor improvement after treatment [55]. After neuropathological analysis of the lesioned rats, no differences in surviving nigral dopaminergic neurons or striatal dopaminergic innervation was observed. Therefore, the authors hypothesize that the behavioural improvement resulted from enhanced levels of tyrosine hydroxylase due to *Parkin* overexpression. To test this, the effects of nigral human *PARKIN* overexpression in intact rats was examined. The human *PARKIN* treated striatum contained more dopamine, suggesting that PARKIN enhances nigral dopaminergic neurotransmission rather than exerting any protective effect on the nigrostriatal tract [55]. Increase in PARKIN levels attenuates methamphetamine-induced decreases in striatal tyrosine hydroxylase immunoreactivity in a dose-dependent manner, indicating that PARKIN exerts a neuroprotective effect on striatal dopaminergic terminals upon methamphetamine neurotoxicity [57]. High dosage of methamphetamine causes selective degeneration of dopaminergic terminals in the striatum, sparing other striatal terminals and cell bodies [57]. The overexpression of AAV-mediated α-synuclein decreases the density of dopaminergic axon terminals in the striatum of rats and monkeys, which is ameliorated by co-expression of PARKIN [59]. Moreover, AAV-delivery of *Parkin* is associated with either less accumulation of α-synuclein protein, phosphorylation at serine residue at 129^th^ position or both [59]. AAV-mediated-tau overexpression induced dopaminergic neuron loss, and PARKIN prevented the loss of substantia nigra dopaminergic neurons in tau-induced dopaminergic degeneration model [56]. Studies performed in young transgenic mice overexpressing *Parkin,* specifically in neurons, show improved MPTP-induced mitochondrial impairment in the substantia nigra, while old transgenic mice present decreased striatal α-synuclein [212]. Also, pharmacological strategies exploit PARKIN signalling activation have been tested. Inhibition of ROCK promotes increased recruitment of HK2, a positive regulator of PARKIN, to mitochondria, leading to increased targeting of mitochondria to lysosomes and removal of damaged mitochondria from cells. Furthermore, ROCK inhibitors have neuroprotective effects in a fly PD model [213]. A sign of warning came from the study performed by van Rompuy et al. [58], where administration AAV2/8-CMV-human *PARKIN*, in (healthy, non-lesioned) wild-type rats substantia nigra induced progressive and dose-dependent dopaminergic cell death, starting from 8 weeks after injection. The authors excluded non-specific cell death induced by an inflammatory response due to the vector preparations. Interestingly, administration of the same vector and dose in mouse substantia nigra did not cause toxicity [58]. The evidence gathered seems to support the use of *PARKIN* viral delivery for the treatment of PD. However, most of these studies were performed in acute and induced disease models, where treatment is often provided before the injury. To the best of our knowledge, there is no direct evidence of functional rescue via viral-mediated delivery of *Parkin* in a *Parkin*-deficient animal. Moreover, although some of these studies show behavioural improvements and dopaminergic neuronal survival, very little is described about the mechanism underlying these observations. The concerns raised by van Rompuy et al. [58] suggest the necessity of performing toxicity assays to study the potential deleterious effect of long term overexpression of *PARKIN,* especially in human-derived tissues.

Overexpression of *PARKIN* has been also exploited as a treatment for AD. In fact, overexpression of *Parkin* ameliorates impaired mitophagy and promotes the removal of damaged mitochondria in amyloid β-treated cells, indicating that upregulation of PARKIN-mediated mitophagy may be a potential strategy also to treat AD [214]. However, not only *PARKIN* gene therapy vectors have been developed and tested. In the literature, there is at least one study assessing the potential of *PINK1* gene augmentation as a treatment for AD. The rationale for that originates from the fact that in the brains of patients with AD and transgenic AD mice model PINK1 is downregulated [60]. AAV-*PINK1* transduction significantly reduced human amyloid-β levels by 65–70% in the hippocampus of transgenic mAPP mice that overexpress a human mutant form of APPbearing both the Swedish (K670N/M671L) and the Indiana (V717F) mutations (APPSwInd) at 11–13 months of age. *PINK1* overexpression promotes the clearance of damaged mitochondria by augmenting autophagy signalling via activation of autophagy receptors (OPTN and NDP52), thereby alleviating amyloid-β-induced loss of synapses and cognitive decline in mAPP mice [60]. Transgenic mice overexpressing the *PARKIN* in neurons were crossed with APP/PS1 transgenic mice. Overexpression of *PARKIN* restored activity-dependent synaptic plasticity and rescued behavioural abnormalities. Moreover, overexpression of *Parkin* was associated with down-regulation of APP protein expression, decreased β-amyloid load and reduced inflammation [215].

A recent study demonstrated that overexpression of *Parkin* cDNA driven by a CMV promoter, encapsulated in AAV2/2, and delivered by intravitreal injection, improved the outcome in a rat model of glaucoma. Delivery of *Parkin* into the retina protected against retinal ganglion cell loss, attenuated glial fibrillary acidic protein (GFAP) expression, promoted optineurin expression, improved mitochondrial health, and partially restored dysfunction of mitophagy in chronic hypertensive glaucoma rats [30].

Khalil et al. [48] studied the impact of *PINK1* overexpression in a *Drosophila* model of HD. Their data demonstrate that *PINK1* overexpression rescues HD neuronal pathology, ameliorated ATP levels, neuronal integrity and adult fly survival, demonstrating that PINK1 counteracts the neurotoxicity of mutant Huntingtin [48]. PINK1 neuroprotection against mutant Huntingtin is dependent on PARKIN, mitofusin and the voltage-dependent anion channel [48].

## Conclusions

The fast-increasing list of scientific publications related to PINK1/PARKIN signalling demonstrates how limited is our knowledge about this pathway and at the same time how disease-relevant this seems to be. It is becoming clear that PINK1 and PARKIN related processes are capable of modulating neurodegeneration and neuroinflammation, either by removing dysfunctional mitochondria, controlling mtDNA release or promoting neuroprotective and anti-inflammatory phenotypes.

Based on the studies here compiled gene augmentation of PARKIN and PINK1 seems a promising strategy for the treatment of brain and retinal neurodegenerative disorders. All the pre-clinical studies summarized in this review not only increase our knowledge about PINK1/PARKIN signalling but raise hope for the development of new treatments for neurodegenerative disorders.

## Data Availability

Not applicable.

## Abbreviations

ΔΨm: Mitochondrial membrane potential
AAV: Adeno-associated virus
AD: Alzheimer’s disease
ADP: Adenosine diphosphate
AICD: APP intracellular domain
AIMP2: Aminoacyl-tRNA synthetase complex interacting multifunctional protein-2
ALS: Amyotrophic lateral sclerosis
AMD: Age-related macular degeneration
AMP: Adenosine monophosphate
APP: Amyloid precursor protein
ARJP: Autosomal recessive juvenile parkinsonism
ATG12: Autophagy related gene 12
ATG101: Autophagy related gene 101
ATP: Adenosine triphosphate
cI-V: Protein complexes (I-V)
cGAMP: Cyclic guanosine monophosphate–adenosine monophosphate
cGAS: Cyclic guanosine monophosphate–adenosine monophosphate synthase
ClpXP: Caseinolytic mitochondrial matrix peptidase
CMV: Cytomegalovirus
CTD: C-terminal domain
dKO: Double knockout
Drp1: Dynamin-related protein 1
dsDNA: Double-stranded DNA
ERRα: Estrogen-related receptor α
ETC: Electron transport chain
FIP200: FAK family kinase-interacting protein of 200 kDa
GBA: Genes including glucocerebrosidase
GFAP: Glial fibrillary acidic protein
GMP: Guanosine monophosphate
HD: Huntington’s disease
HTT: Huntingtin
IBR: In-between-RING
IMM: Inner mitochondrial membrane
IOP: Intraocular pressure
iNOS: Inducible nitric oxide synthase
KO: Knockout
LC3: Microtubule-associated protein 1A/1B-light chain 3
LPS: Lipopolysaccharide
MCP-1: Monocyte chemoattractant protein-1
MDV: Mitochondrial derived vesicles
MHC: Major histocompatibility complex
OMM: Outer mitochondrial membrane
MPP: Mitochondrial processing peptidase
MPTP: 1-methyl-4- phenyl-1,2,3,6-tetrahydropyridine
mtDNA: Mitochondrial DNA
MTS: Mitochondrial targeting sequence
NDP52: Nuclear dot protein 52
NFE2L2: Nuclear Factor, Erythroid 2 Like 2
NO: Nitric oxide
NRF-1: Nuclear respiratory factor-1
NT: N-terminal regulatory domain
OPA1: Optical atrophy-1
OPTN: Optineurin
OXPHOS: Oxidative phosphorylation
PACRG: Parkin co-regulated gene
PARL: Presenilins-associated rhomboid-like protein
PARKIN: Parkin RBR E3 ubiquitin protein ligase
PARP1: Poly(ADP-ribose) polymerase-1
PD: Parkinson’s disease
PGAM5: Phosphoglycerate mutase family member 5
PGC-1α: Proliferator-activated receptor gamma coactivator-1α
PGK: Phosphoglycerate kinase 1
Pi: Inorganic phosphate
PINK1: PTEN-induced kinase 1
PKD: Protein kinase domain
PPARγ: Proliferator-activated receptor gamma
PtdIns3K: Phosphatidylinositol 3-kinase
PSEN1: Presenilin 1
PSEN2: Presenilin 2
RAB: RAS-related GTP-binding
RBR: RING1-IBR-RING2
REP: Repressor element of Parkin
ROS: Reactive oxygen species
SOD1: Superoxide dismutase 1
STING: Stimulator of interferon genes
Syt11: Synaptotagmin-11
TBC1D9: TBC1 domain family member 9
TBK1: TANK Binding Kinase 1
TIM: Translocase of the inner membrane
TM: Transmembrane domain
TOM: Translocase of the outer membrane
TDP-43: Transactive response DNA-binding protein 43 kDa
Ubl: Ubiquitin-like
UBR: Ubiquitin binding region
ULK1: Unc-51 like kinase 1
ZNF746: Zinc finger protein 746
